# Ultra-fast responsive colloidal–polymer composite-based volatile organic compounds (VOC) sensor using nanoscale easy tear process

**DOI:** 10.1038/s41598-018-23616-8

**Published:** 2018-03-28

**Authors:** Hyung-Kwan Chang, Gyu Tae Chang, Ashish K. Thokchom, Taesung Kim, Jungyul Park

**Affiliations:** 1 Department of Mechanical Engineering, Sogang University, 35 Baekbeom-ro (Sinsu-dong), Mapo-gu, Seoul 04107 Korea; 2 Department of Mechanical Engineering, Ulsan National Institute of Science and Technology (UNIST), 50 UNIST-gil, Ulsan, 44919 Republic of Korea; 3 Interdisciplinary Program of Integrated Biotechnology, Sogang University, 35 Baekbeom-ro (Sinsu-dong), Mapo-gu, Seoul 04107 Korea

## Abstract

There is an immense need for developing a simple, rapid, and inexpensive detection assay for health-care applications or monitoring environments. To address this need, a photonic crystal (PC)-based sensor has been extensively studied due to its numerous advantages such as colorimetric measurement, high sensitivity, and low cost. However, the response time of a typical PC-based sensor is relatively slow due to the presence of the inevitable upper residual layer in colloidal structures. Hence, we propose an ultra-fast responsive PC-based volatile organic compound (VOC) sensor by using a “nanoscale easy tear (NET) process” inspired by commercially available “easy tear package”. A colloidal crystal-polydimethylsiloxane (PDMS) composite can be successfully realized through nanoscale tear propagation along the interface between the outer surface of crystallized nanoparticles and bulk PDMS. The response time for VOC detection exhibits a significant decrease by allowing the direct contact with VOCs, because of perfect removal of the residual on the colloidal crystals. Moreover, vapor-phase VOCs can be monitored, which had been previously impossible. High-throughput production of the patterned colloidal crystal–polymer composite through the NET process can be applied to other multiplexed selective sensing applications or may be used for nanomolding templates.

## Introduction

Volatile organic compounds (VOCs) are toxic to the human body and harmful to the environment as some of the VOCs form the main component of photochemical smog and industrial pollution^1–4^. The most conventional methods for VOC detection include mass spectrometry and gas chromatography^5,6^. However, although these methods allow high-precision and high-resolution detection of VOCs, they have a long measuring period, bulky size, and high cost, which restrict these methods from onsite monitoring and real-time analysis. A VOC sensor made of polydimethylsiloxane (PDMS) is an alternative to overcome these disadvantages^7^. PDMS is widely used in the microfluidics field and integrated circuit packaging application^8^, but has recently received increasing attention for a VOC sensing application due to its swelling effect upon absorbing organic solvents^9^.

In this study, we propose an ultra-fast responsive photonic crystal (PC)-based colorimetric sensor made of colloidal crystal–PDMS composite by using the PDMS swelling effect, which shows a rapid structural color change when exposed to VOCs. The PC enables the control and manipulation of photons through artificially designed periodic structures and is intensively used as a colorimetric sensor to detect biomolecules^10^, humidity^11,12^, temperature^13^, ionic strength^14^, pH^15^, and mechanical force^16^. However, the previously reported PC sensors for chemical detection suffer from a slow response time due to the upper residual on the crystallized colloids, which is inevitable in the previously proposed fabrication process. The colloidal crystal–PDMS composite VOC sensor exhibits a 6 nm bandgap shift when exposed to ethanol for 10 min^7^. The colloidal crystal–gel composite humidity sensor requires 1.5 h for the bandgap shift measurement^11^. In contrast, inverse opal^17^, block-copolymer^18^, and porous structures^19^ have fast response time within a few seconds. However, they require the limited materials synthesized with the specific process, or complex fabrication processes and relatively long processing time, or are severely influenced by untargeted molecules like humidity.

**Figure 1:**
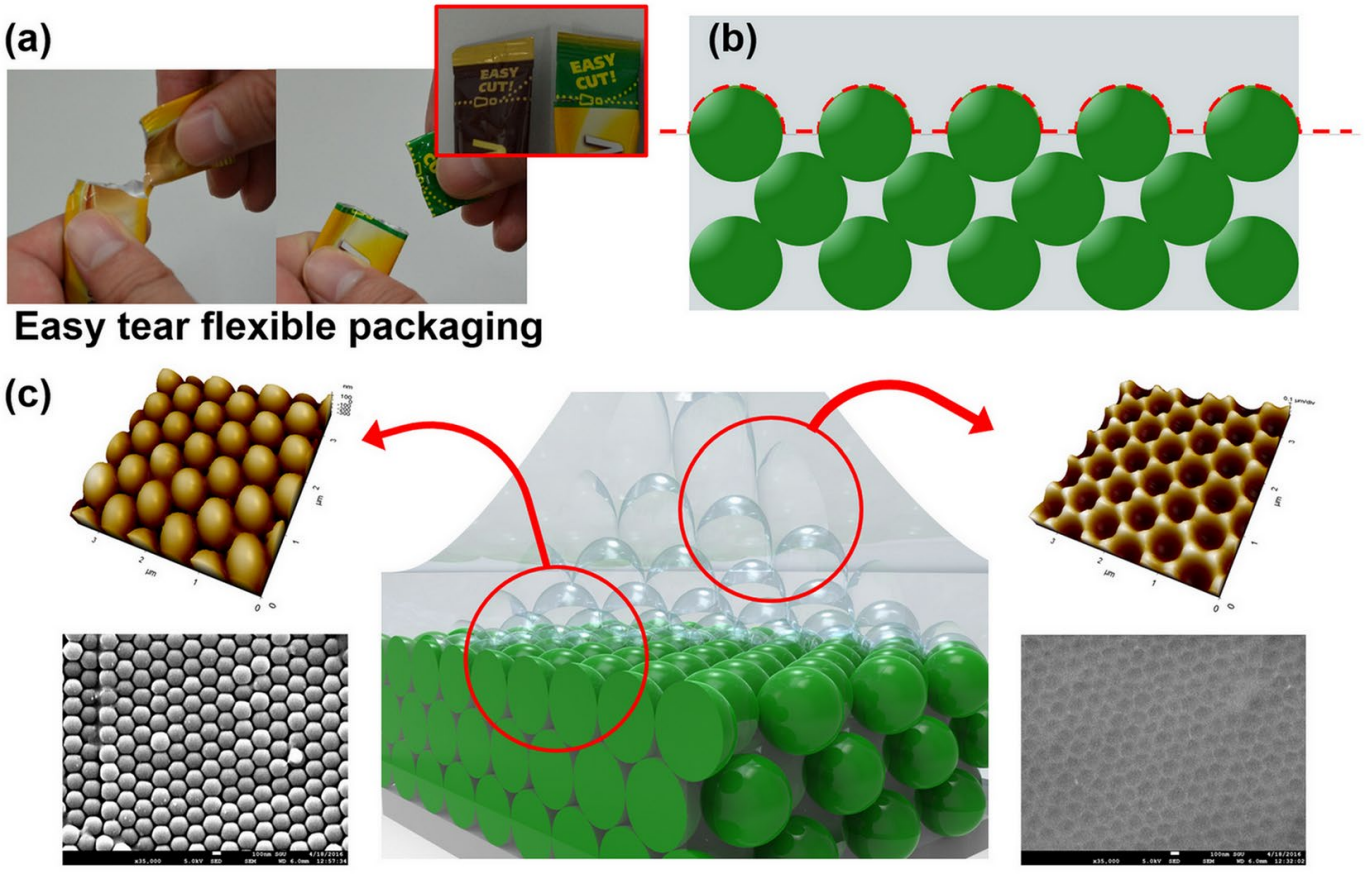
Concept of nanoscale easy tear (NET) process inspired by (**a**) commercially available easy tear packaging. Colloidal crystal–PDMS composite is obtained without any residual on colloidal structures due to (**b**) tear propagation. (**c**) SEM and AFM image of colloidal crystal–PDMS composite and upper bulk PDMS layer clearly demonstrate the success of the NET process.

Easy tear packaging is widely used in the flexible packaging industry because of its convenient and clean-tear capabilities, which facilitates a consumer accesses the product, as shown in Fig. 1a. Score lines are normally created by laser scoring to realize a narrow channel in the material for a tear to follow. Here, a colloidal crystal–PDMS composite that enables direct contact with the target organic compounds is also constructed by a “nanoscale easy tear (NET) process” inspired by easy tear packaging. Tear propagation allows a perfect exfoliation of the upper PDMS layer without any residual on the crystallized colloidal surface (Fig. 1b), which shows a rapid swelling of the PDMS among the colloidal structures in contact with VOCs. This direct contact of colloidal crystal-PDMS composite with target molecules can resolve the typical problem of slow response time of PC sensor and also make it possible to detect vapor-phase VOCs, which was nearly difficult in the previous PC sensor.

Since the swelling property of PDMS is depend on the solubility of solvent^9^ and the swelling ratio is different with respect to kinds of polymer even in the same solvent, the patterning of colloidal crystal-polymer can be used for detecting the concentration of solvent or selective sensing the mixture of solvent. In this study, variously patterned 3D colloidal crystal–PDMS composites, including simple cuboid as well as dome-shaped patterns, are successfully fabricated through wettability contrast patterning^20^ or inkjet printing^21^. We demonstrated this patterned array as a multiplex sensor for figure out the concentration of ethanol and the mixtures of various solvents. The additional application of the NET process was demonstrated using other polymer materials, namely a colloidal crystal–hydrogel composite as a humidity sensor with a rapid response time.

The proposed NET process is differentiated from a typical micromolding process because the main goal of the process is the high-throughput production of the colloidal crystal–PDMS composite via tear propagation. However, in case of micromolding, the demolded PDMS part is the main product that is utilized for the microchannel or microstamp.

## Results and Discussion

The colloidal crystal-PDMS composite based VOC sensor could be made by the NET process during peeling off bulk PDMS. The successful NET process (the perfect removal of residual of colloidal crystal-PDMS composite) is owing to the exceptionally concentrated stress at the interface of outer surface of colloidal-PDMS and bulk PDMS. In order to explain this concentrated stress and how forces and physical properties contribute to induce it, numerical simulation via COMSOL Multiphysics (COMSOL Inc., Seoul, Korea) was performed. The detailed description of the simulation model is described in the supplementary section. The simulation model consists of three layers of nanoparticles and PDMS. It is assumed that the colloidal particles are assembled together by van der Waals force (Fig. S1a)^22^. Therefore, the van der Waals force is applied at the colloid particle domain by using a body load condition in the simulation. The bottom layer of the assembled nanoparticles is assumed to be fixed at the substrate firmly, and the prescribed displacement condition is applied to the top boundary in the normal and shear directions.

**Figure 2:**
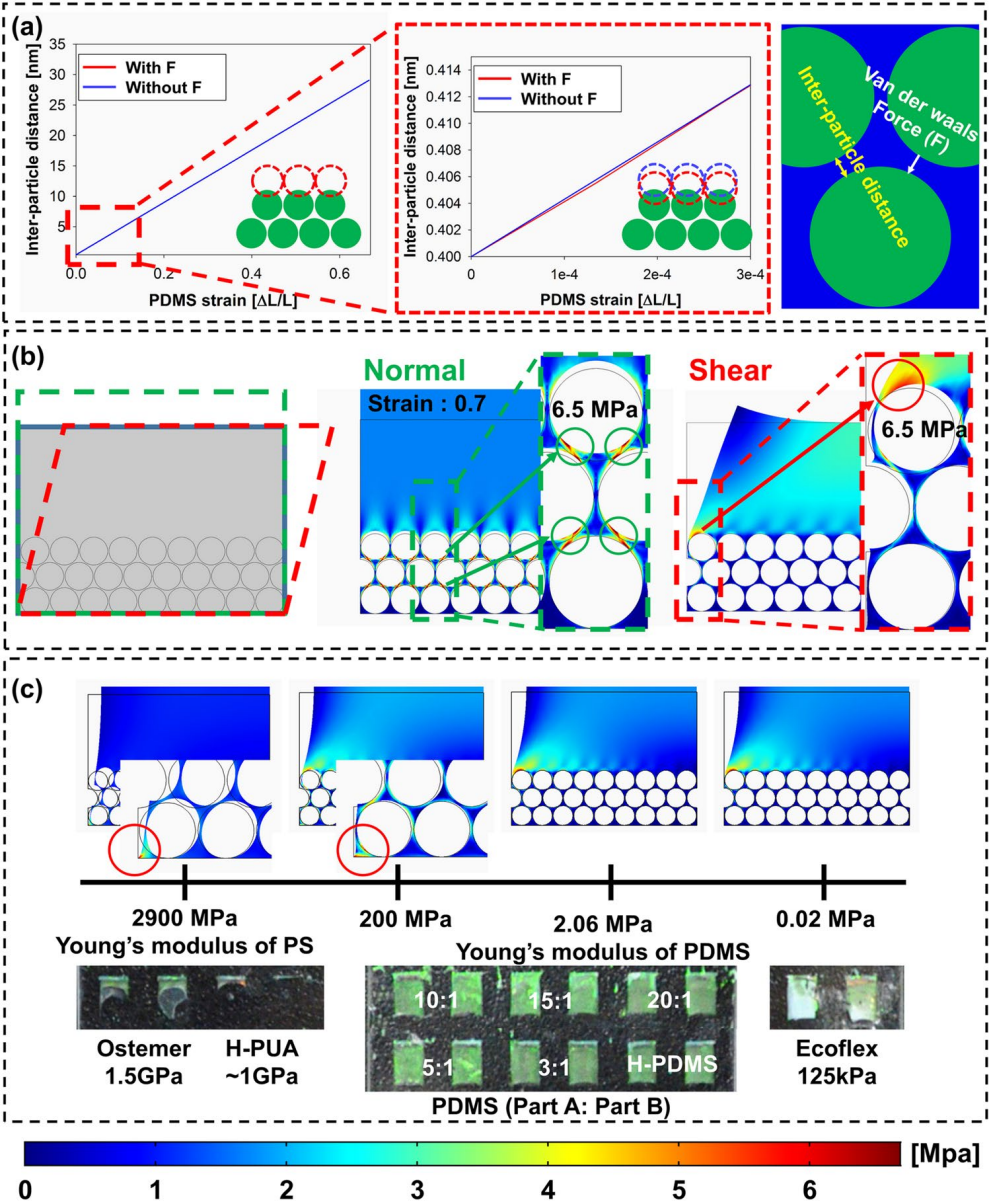
Numerical simulation for explaining the mechanism of the nanoscale easy tear (NET) process. (**a**) Variation of inter-particle distance with and without van der Waal force according to the increases of the normal displacement of PDMS. (**b**) Von Mises stress plot with respect to bulk PDMS displacement in the normal and shear directions. (**c**) Von Mises stress plots and experimental results of the peel-off process depending on various Young’s modulus of polymer.

As shown in Fig. 2a, the van der Waals force works only in the initial state under normal displacement. However, the van der Waals force rapidly converges to zero according to the increase of inter-particle distance under the increase of normal displacement. Furthermore, the maximum van der Waals force is too low to maintain the original positions of nanoparticles. When the strain of the PDMS reaches 0.7, homogenous stress distributions corresponding the ultimate tensile strength of PDMS (6.25 MPa)^23^ occur at all interfaces of nanoparticles as shown in Fig. 2(b). This homogenous stress distribution make it difficult to predict the initial crack location. That is, we cannot achieve the exceptional stress concentration on the first layer under the normal directional displacement. In contrast, under the shear deformation of the upper PDMS, the concentrated ultimate tensile strength occurs at the first layer of particles only. The simulation results indicate that the shear stress is a key player in the NET process due to asymmetry. In the actual NET process, the shear stress occurs naturally when the bulk PDMS is peeled off using forceps. Therefore, to mimic this real situation, another simulation model for the boundary position in the colloidal crystal-PDMS was developed, which was similar to the previous one. The different point is that the symmetric boundary condition was imposed at one side, and the free boundary at the other side. Then, the normal displacement was applied to bulk PDMS. As shown in Fig. S1(b), the concentrated stress was found at the first layer of colloidal crystal-PDMS, which is similar to the simulation results under shear deformation. The middle position of colloidal crystal-PDMS is similar situation to the previous simulation under the normal displacement (Fig. 2(b)). These simulation results were experimentally verified by observing the exfoliated PDMS layer and the remaining crystallized nanoparticles (SEM and AFM images in Figs 1c and 4, respectively). The images clearly illustrate that the upper bulk PDMS layer is completely removed from the surface of the colloidal crystal without residual.

The effect of mechanical property in the polymer was also investigated in the NET process using numerical simulation (Fig. 2(c)). Young’s modulus of polystyrene is 1.9~2.9 GPa and it is 1000 times higher than that of the PDMS. When the shear stress is applied, the PS particle is not deformed which produces the concentration of stress at the first layer of PCs. However, if the Young’s modulus of polymer is increased up 200 MPa, the deformation of PC structure happens and the concentration of stress exhibit at the bottom layer of colloidal-polymer composite. It means the colloidal-polymer composite with high mechanical stiffness may be easily separated from the substrate before the start of tear propagation. To prove this, we conducted the peel-off process using various polymers with Young’s modulus of 200 kPa to 1.5 GPa. As expected from the simulation, when polymers with high Young’s modulus was used, the colloidal-polymer composite was separated with bulk PDMS from the bottom layer, and it means the fail of NET process. In case of polymers with Young’s modulus of less than 200 MPa, the colloidal-polymer composite was realized successfully. From the simulation, we could conclude that the successful NET process clearly depends on the shear deformation and the difference of mechanical stiffness between the hard nanoparticles and the soft polymer.

**Figure 3:**
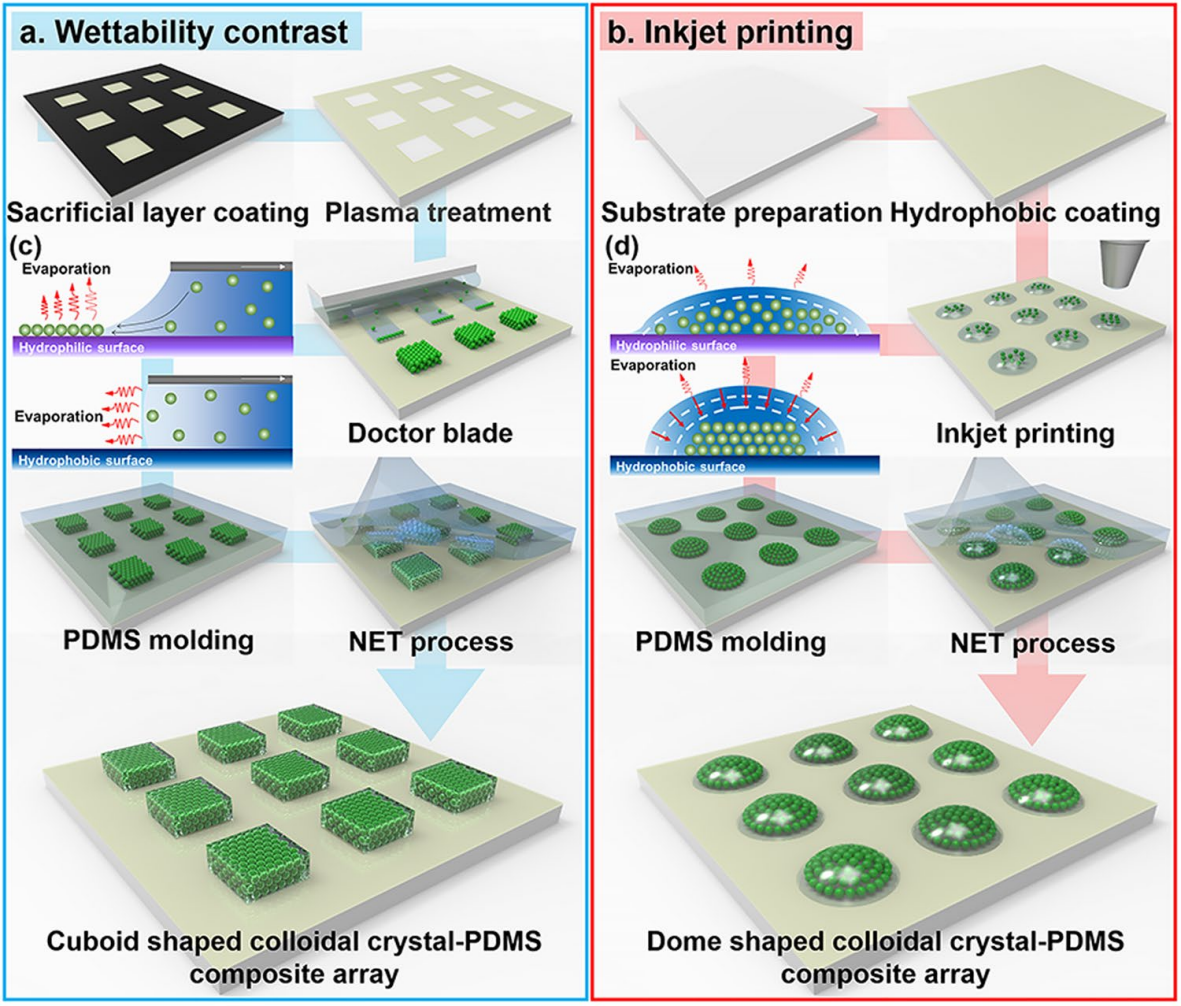
Fabrication process for creating a colloidal crystal–PDMS composite array by using (**a**) wettability contrast or (**b**) inkjet printing. (**c**) Schematic of the convective assembly with wettability contrast on hydrophilic or hydrophobic patterned areas. (**d**) Assembly of schematic particles within the ink droplet.

The fabrication process for the colloidal crystal–PDMS composite VOC sensor is depicted in Fig. 3. Accurate patterning of colloidal particles in 2D or 3D structures has attracted considerable interest because of its promising potential for a multiplex PC-based colorimetric sensor array^24–26^. Patterning a colloidal crystal using wettability contrast is preferred due to a simple processing step^20^. Here, the wettability contrast was realized by a localized plasma-induced conversion of surface functional groups on the self-assembled monolayer (SAM). When the self-assembled octadecyltrichlorosilane (OTS) monolayer, which possesses a hydrophobic property, is exposed to oxygen plasma, it causes hydroxylation and oxidation of the methyl end groups on the OTS-covered substrate and the plasma-modified region results in a hydrophilic area^27^. A cuboid 3D colloidal crystal pattern is successfully created by the wettability contrast, which confines the colloidal dispersion solution to the designed substrate area (Fig. 3a).

In this study, the crystallization of the nanoparticles is accomplished by a doctor blade method based on convective assembly^28^. As shown in Fig. 3c, a convection assembly occurs while wetting a substrate with a contact angle lower than ~20°, which indicates that the thickness of the solvent layer equals the particle diameter. An increase in the particle layer results in the simultaneous withdrawal of the substrate. If the withdrawal velocity is equal to the crystallization formation rate, then the homogeneous colloidal crystal layer grows continuously. With respect to the steady-state assembly, a simple equation that describes particle accumulation in the solvent flux and drying region are proposed by Dimitrov and Nagayama^29^ as follows:

1 $$v=\frac{\beta {j}_{e}\phi l}{h(1-\varepsilon )(1-\phi )}$$

This equation establishes relations among the growth velocity of the layer, *v*; porosity, *ε*; height of the assembled layer, *h*; volume fraction ratio of the particles in the suspension, *φ*; a coefficient that relates the solvent velocity to the particle velocity, *β* (0 < *β* < 1); and the evaporation rate of pure water, *j*_*e*_. The variable *l(x)* is defined as *l(x)* = *J*_*evap*_*/j*_*e*_*(x)*, where *J*_*evap*_ denotes the rate of evaporation averaged over the drying length and *j*_*e*_*(x)* indicates the evaporation rate withdrawn at any point of the drying region. This equation shows that three key process parameters, including substrate velocity, particle volume fraction, and solvent evaporation rate, can be used to control the coating thickness and structure. With respect to fixed evaporation rates and volume fractions, the thickness of the colloidal crystal layer is manipulated by controlling the substrate speed. As previously mentioned, the assembly commences when the solvent layer thickness in the hydrophilic region is equal to the particle size, and the particles are assembled into a close-packed structure. However, in the hydrophobic region, the thickness of the solvent layer is never close to the particle diameter and the lateral capillary force prevents crystallization.

The dome-shaped colloidal crystal is fabricated by inkjet printing of the colloidal solution on the hydrophobic surfaces (Fig. 3b). Convective assembly involving the use of low-adhesive hydrophobic substrates results in the formation of a hemispherical dome structure due to the continuously retreating three-phase contact lines while keeping the contact angle unchanged during the evaporation process (Fig. 3d)^21^. Thus, the dome-shaped colloidal crystals are prepared by inkjet printing of a colloidal solution on a hydrophobic surface.

The VOC sensors based on a colloidal crystal–PDMS composite are fabricated by embedding PDMS material into colloidal crystals via the NET process. The PDMS swells in a non-polar organic solvent and the swelling rate depends on the solvent polarity. Solvents with solubility parameters similar to PDMS generally swell the PDMS more than solvents with solubility parameters that are substantially different from those of the PDMS^9^. The PDMS mixture is poured on the colloidal crystals, and the pores between the polystyrene nanoparticles are completely filled with the premixed elastomer of PDMS through capillary action. The complete filling time, *t*, is estimated as follows^30^:

2 $$t=\frac{2{\eta }_{s}h}{R\gamma \,\cos \,\theta }$$

where *η*_*s*_ denotes the viscosity of the liquid epoxy resin, *R* denotes the hydraulic radius of the nanopore, *γ* denotes the surface tension at the liquid/air interface, and *θ* denotes the contact angle. For example, when the nanoparticle size is 220 nm (i.e., R ~ 33 nm, the nano-interstices between close-packed nanoparticles constitute 15% of the sphere size), height of the assembled layer *h* = 2.6 μm, the viscosity *η*_*s*_ is ~4000 cps at 25 °C, the surface tension *γ* is ~19.8 mN/m, and the contact angle is 70°^31^, then the complete filling time of PDMS is approximately estimated as 26.6 s. To remove the upper PDMS layer on the crystallized nanoparticles after curing, the PDMS substrate is gently peeled off using forceps with proper shear stress, and the upper PDMS layer is then successfully exfoliated through tear propagation.

**Figure 4:**
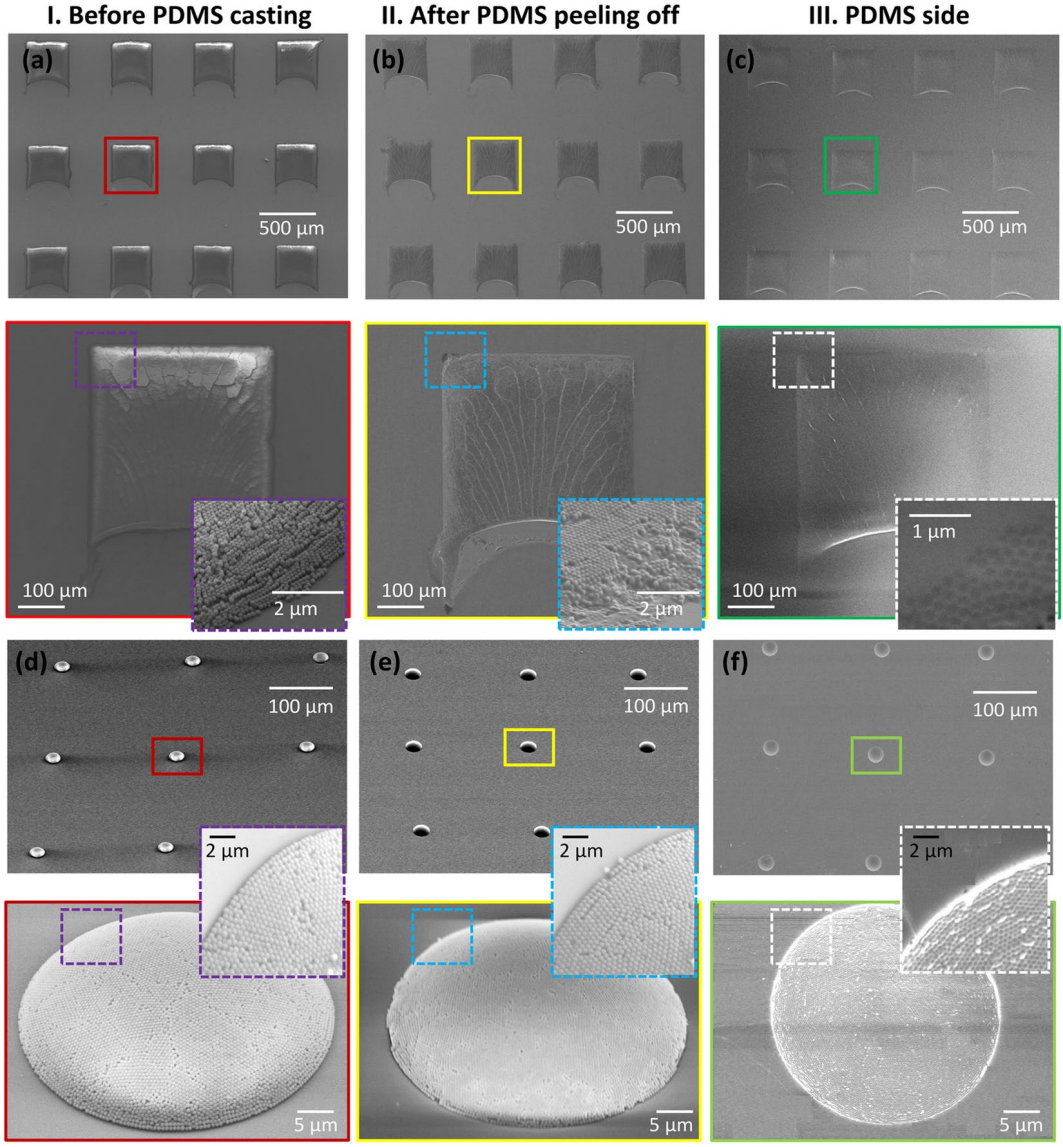
SEM Image of (**a**–**c**) cuboid and (**d**–**f**) dome-shaped (I) colloidal crystal (II) colloidal crystal–PDMS composite (III) exfoliated PDMS. Each inset represents a magnified image.

As shown in Fig. 4, different patterns of 3D colloidal crystal–PDMS composites are fabricated and include a simple cuboid as well as a dome-shaped pattern, through wettability contrast patterning or inkjet printing. The colloidal crystals are patterned on a hydrophilic/hydrophobic patterned substrate to produce a simple cuboid pattern. When the nanoparticle suspension is deposited on the wettability control substrate, the droplet forms a circular contact line and the radius of the contact line is half the width of the hydrophilic pattern. Due to the wettability contrast, the convective assembly is only initiated in the hydrophilic region. As a result, a simple cubic colloid crystal is formed and is circular at the bottom of the cube, as shown in Fig. 4a. The average thickness of the crystallized colloid at a moving speed of 0.01 mm/s is 2.6 μm, as measured with a profilometer (Dektac, Bruker Inc., USA).

As previously mentioned, a hydrophobic substrate is used in inkjet printing to fabricate a dome-shaped pattern. The hydrophobic nature of the substrate can induce a secondary fluid flow, known as Marangoni flow, inside the injected droplet by the inkjet printer. The Marangoni flow also enhances the formation of close-packed particles and prevents the unwanted coffee-ring-like deposition behavior of drying droplets^32^. Nanoparticles in the droplet successfully form dome-shaped nanostructures (Fig. 4d). Figure 4b and e depict the colloidal crystal–PDMS composites on the substrate, and Fig. 4c and f show the surface images of the exfoliated PDMS of cuboid and dome shapes, respectively. The colloid crystal pattern is clearly observed on the exfoliated PDMS side. Additionally, various nanoparticle sizes were tested for realizing colloidal crystal–PDMS composites through the NET process, as shown in Fig. S2a. The exfoliated PDMS layer may be used as a master for replica molding to generate the structures with nanotopological structures for other applications. Furthermore, in order to confirm a high-throughput and large-scale production of the patterned colloidal crystal-polymer composite, a large areal colloidal crystal array on a whole wafer was tested, which was formed by the inkjet printing (Fig. S2b). Statistical analysis was performed by imaging processing for 4 wafer samples to investigate how many colloidal-PDMS composites are successfully obtained through the NET process. Here, we found the successful yield for the NET process was over 95% without any damage for the prepatterned colloidal crystal array. From this result, it is concluded that the colloidal crystal–PDMS composites can be constructed in a high-throughput manner.

**Figure 5:**
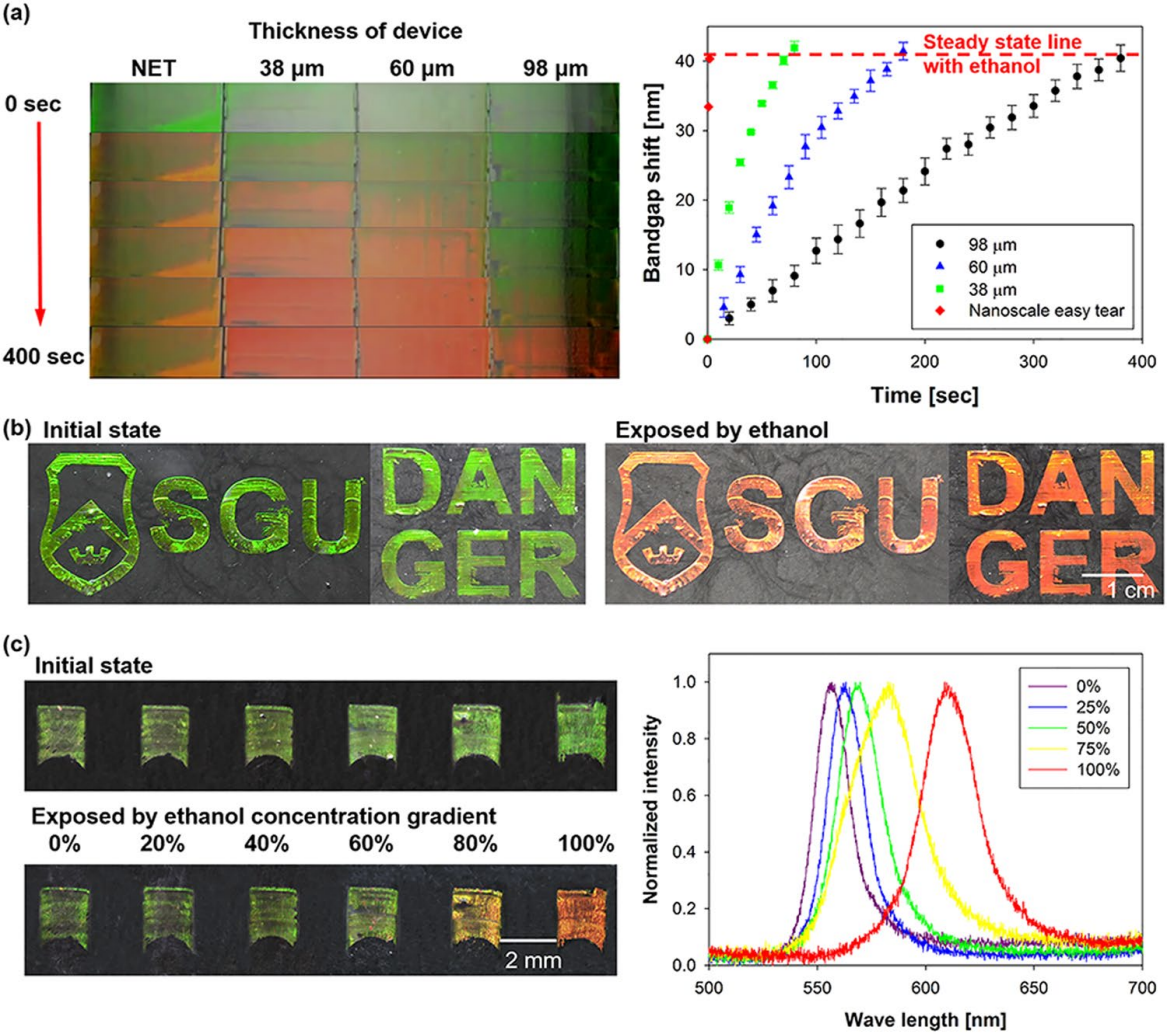
(**a**) Color change and bandgap shift relative to thickness of the upper PDMS layer from the crystallized nanoparticles. (**b**) Color change in the graphical patterned colloidal crystal–PDMS composite sensor due to stimulation with ethanol. (**c**) Color change in 6 × 1 array pattern and bandgap shift relative to the ethanol concentration.

The colorimetric detection of various organic solvents is performed using the fabricated sensors. Diffractions that fall into the visible range are usually preferred to use PCs as sensors because the optical output is directly observed by the naked eye without requiring complicated and expensive apparatus to read the signals. The initial peak wavelength of the fabricated colloidal crystal-based chemical sensor is ~550 nm (shown in green in Fig. 5a) and is predicted using the Bragg equation. The Bragg equation for normal incidence is as follows^33^:

3 $$\lambda =2d{n}_{eff}$$

Where *λ* is the peak wavelength of reflected light, *d* = 0.816, *D* denotes the interlayer spacing in the (111) direction of the FCC structure, *D* = 220 nm represents the diameter of a nanoparticle, and *n*_*eff*_ denotes the effective refractive index of the sample. The effective refractive index of a two-phase structure is estimated as follows:

4 $${n}_{eff}=f{n}_{PS}+(1-f){n}_{PDMS}$$

where *f* = 0.74 denotes the void fraction of the porous structures for an ideal FCC structure, and *n*_*PS*_ = 1.57 and *n*_*PDMS*_ = 1.49 represent the refractive indices of polystyrene and PDMS materials, respectively. When the chemical sensor is immersed in a non-polar organic solvent, the interlayer spacing (*d*) of colloidal crystal increases as the PDMS matrix swells (Fig. S3a). The swelling ratio of PDMS varies depending on the solvent. Generally, the solubility parameter is useful for predicting the swelling behavior of the PDMS in a solvent. According to the Hildebrand-Scatchard equation^34^, solvents with solubility similar to that of PDMS can swell PDMS effectively. To be consistent with this fact, the bandgap shift exhibited a significant change in the order of methanol, ethanol, and acetone. The color change of the NET-processed sensor is excessively fast, and thus, it is difficult to observe the progress of color change. Thus, the sensor with 98 μm thickness is used to observe the progress of color change clearly as shown in Fig. S3b. In the case of methanol and ethanol, the color change, as confirmed by the naked eye, is observed after ~3 min and reaches a steady state in ~10 min. At the steady state, the band gap shifts are 30 nm and 40 nm, respectively. The color change by acetone is confirmed by the naked eye in ~5 s and reaches a steady state with a bandgap shift of 125 nm in 20 s. In case of methanol and ethanol, the same bandgap shift values (methanol: 30 nm, ethanol: 40 nm) were observed with and without the NET process but much faster response time (<2 s) was found for the NET-processed sensor (Fig. S3c). Furthermore, the similar response time within 2 s was observed even if the pattern size was changed (Fig. S4).

To quantitatively analyze the improved response time by using the proposed method, we measure the bandgap shift time according to thickness of residual when the sensor is exposed to the ethanol solvent. Figure 5a shows the bandgap shift time based on the thickness of the upper PDMS layer. With respect to the control groups, the various upper PDMS layers (residual layers) are created by changing the spin-coating speed. The thicknesses of the residual PDMS correspond to 38, 60, and 98 μm corresponding to spin speeds of 1000, 2000, and 3000 rpm, respectively. The response time is dependent on the thickness of the residual. A decrease in the thickness of the upper PDMS layer decreases the response time. The bandgap shifts of the colloidal crystal–PDMS composite VOC sensor reach 40 nm (the steady-state value) within 2 s when exposed to ethanol. This is more than 3000 times faster than the previously reported results^7^. Additionally, when the chemical sensor is removed from the organic solvent and completely dried in air, the PDMS matrix retracts and returns to its original position (Movie S1a). In order to enable easier recognition of a dangerous situation, the colloidal–PDMS composite sensor was graphically patterned as shown in Fig. 5b. The graphically patterned sensor also turns from green to red following stimulation with ethanol (Movie S1c).

The range of color change is determined not only depending on the solubility of the solvent and PDMS, but also on the concentration of the solvent in the mixture. Previous studies have shown that as the fraction of ethanol in water increases, bandgap shift increases and this result was not linear^35^. In this study, we fabricate a simple cuboid 6 × 1 array to detect the concentration change of the solvent. Figure 5c shows the variation of color in the patterned devices when exposed to the ethanol gradients. This result shows that the patterning colloidal-PDMS composite can be used to figure out the concentration conditions by the color change. Additionally, it is possible to catch different solvent mixtures selectively by patterning the colloidal-PDMS and colloidal-other polymers having different swelling properties about PDMS (Fig. S5). Here we exposed various solvent mixtures to colloidal-PDMS, colloidal-h-PDMS, and colloidal-Ecoflex composites. In the case of h-PDMS or Ecoflex, no color change was observed in the water-ethanol mixture, whereas PDMS showed a clear orange-yellow color. In the water-acetone mixture, all polymers showed the red-shift, but the different visible colors were observed: PDMS was red, h-PDMS orange, and Ecoflex yellow. Also, when exposed to the water-ethanol-acetone mixture, the larger color change was observed, so that PDMS and h-PDMS reached almost infra-red. For this reason, we expect that the patterning different colloidal-polymer composite would be a solution for selective detection in VOC sensing.

**Figure 6:**
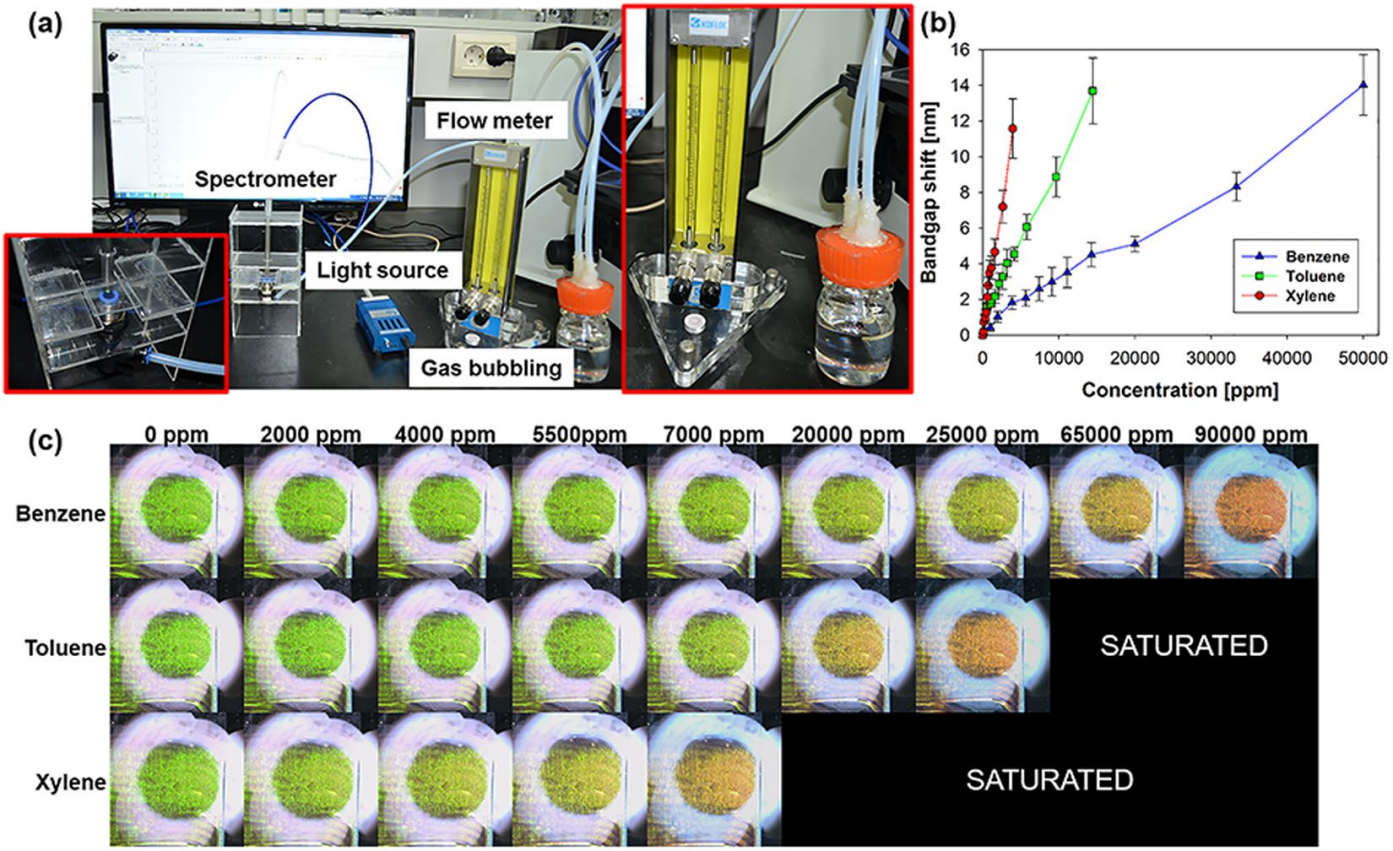
(**a**) Experimental setup used for vapor phase analyte generation and concentration adjustments. (**b**) Bandgap shift of NET-processed sensor by stimulating vaporized VOCs. (**c**) Color change based on the analyte concentration.

The colloidal crystal–PDMS composite VOC sensor exhibits different bandgap shifts based on the types of solvents. Ethanol, methanol, and acetone have a relatively low PDMS expansion ratio. However, high PDMS swelling ratios in BTX solvents make it difficult to observe the bandgap shift because the shifted wavelength exceeds the visible region and the shift is excessively fast. Hence, a device fabricated by the NET method sensitively detects BTX solvents that are harmful to human health. Therefore, the presented VOC sensor works even if it is exposed to vapor-phase VOCs (Movie S1b). The devices are tested to detect vapor-phase analytes at a room temperature of 24 °C and atmospheric pressure, as shown in Fig. 6a. To measure bandgap shift time, the sensor is placed in a gas chamber and directly exposed to the gas. An increase in the analyte concentration leads to the observance of a higher bandgap shift in the order of xylene, toluene, and benzene (Movie S2 and Fig. 6b). The results of the bandgap shift are measured using a spectrometer, as shown in Fig. 6c. The device sensitivity for detecting benzene, toluene, and xylene corresponds to 1601.3, 782.8, and 289.1 ppm/nm, respectively. These results also well match the order of the solubility parameters^9^.

**Figure 7:**
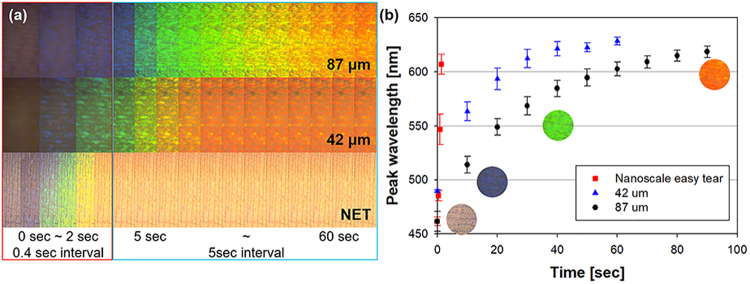
(**a**) Color change (**b**) and bandgap shift with respect to thickness of the upper acrylamide hydrogel layer when the device is exposed to humidity.

The above results show that the NET process significantly improves the response speed of the sensor. To demonstrate the possibility of the NET process besides the colloidal crystal–PDMS composite based sensor, a humidity sensor is fabricated using a colloidal crystal-hydrogel (acrylamide) composite. When exposed to moisture, the bandgap shift of the colloidal crystal–acrylamide composite approximately corresponds to 300 nm, and thus, the particle diameter is adjusted from 220 nm to 186 nm to change the initial peak to 450 nm (blue). As a result, the colloidal crystal–acrylamide composite changes color from blue to red when placed in a 100% humidity chamber (Movie S3 and Fig. 7a). In the control group, humidity sensors with thicknesses of 42 and 87 μm are fabricated. The bandgap shift time is observed to be slower when the thickness of the residual layer increases, as shown in Fig. 7b, which is similar to the result of the colloidal crystal–PDMS composite. Generally, hydrogel (Young’s modulus: 2–50 kPa)^36,37^ is brittle and possesses considerably low elasticity when compared to PDMS (Young’s modulus: 2.05 MPa)^23^. Therefore, the success of the NET process of the colloidal crystal–hydrogel composite implies that it can be applied to produce various other ultra-fast responsive colloidal crystal–polymer composite sensors.

## Conclusions

In this study, the NET process, inspired by an easy-tear packaging product, was proposed for high production of colloidal crystal-PDMS composite based VOC sensors. The physical mechanism of the NET process through the crack propagation between the colloidal-PDMS composite and bulk PDMS was quantitatively analyzed with numerical simulation and it revealed that the shear stress and the difference of mechanical stiffness between PS nanoparticle and the surrounded polymer are main contributors. The perfective removal of residual layer of PDMS in the colloidal–PDMS composite made it possible to achieve a significantly fast response time and the detection of vapor-phase to various chemical stimuli. The potential for a multiplex sensing for selective and multiple target detection was successfully demonstrated by patterning the colloidal crystal-polymer. With respect to the patterned colloidal crystals with various topology and graphics, the colloidal crystal–PDMS composite could be constructed in a high-throughput manner through the proposed NET process. The proposed VOC sensor was fabricated with a compact size, low cost, and simple fabrication process and displays great characteristics for easy, intuitive, real-time, and onsite monitoring via colorimetric detection. The NET process presented in this study can offer other opportunities except for VOC sensors because of various colloidal crystal polymers or colloidal crystal–hydrogel composites can be realized via the same manner. More interestingly, the exfoliated PDMS may be used as a master for replica molding for other applications.

## Methods

### Convective assembly

The colloidal crystals were prepared using a doctor blade method based on a convective assembly. Motorized actuators (Z825B, Thorlabs, Inc) and the XYZ-stage were used to control the substrate moving speed. First, the hydrophilic/hydrophobic mold was fixed on the motorized translation stage. A suspension of polystyrene nanoparticles (Polysciences, Warrington, USA) was deposited on a hydrophilic/hydrophobic patterned substrate by using a micropipette. It was covered with a hydrophobic cover glass with OTS functionalized using the SAM method. The gap height between the two substrates was adjusted to 500 μm by the Z-axis stage. The suspension was dried at a substrate moving velocity of 0.01 mm/s while maintaining a relative humidity (RH) of less than 20% by using a dehumidifier. When all the solution evaporated, the substrate was baked at 95 °C for 1 h such that it was completely dry.

### Fabrication of a hydrophobic/hydrophilic template

OTS (CH_3_(CH_2_)_17_SiCl_3_) for the hydrophobic area was purchased from Aldrich (product no. 104817). The cleaned glass slides were immersed in 1 mM OTS/toluene solution for 2 min to ensure that the glass slides were hydrophobic. Given this condition, a complete OTS monolayer was formed on the glass slide. The OTS-treated glass was rinsed with ethanol to remove excess OTS prior to drying. A positive photoresist (AZ GXR-601-46, AZ Electronic materials) was spin-coated as a sacrificial layer material to form a hydrophilic/hydrophobic patterned glass slide and prebaked at 95 °C for 1 min. The PR-coated substrate was exposed to a UV aligner (MA 6, SÜSS MicroTec AG), developed (AZ-300 MIF, AZ Electronic material), rinsed, dried with nitrogen, and hard-baked at 110 °C for 90 s. With respect to the plasma treatment, samples were loaded into a plasma system (CUTE-MP, Femto science) and exposed to oxygen plasma for 2 min. The removal of the sacrificial layer with acetone was followed by preparing a hydrophilic/hydrophobic patterned substrate.

### Inkjet printing

The dome-shaped colloidal crystals were prepared by inkjet-printing a colloidal solution on a hydrophobic surface. A piezoelectric drop-on-demand inkjet printer (DMP-2800, Fujifilm Dimatix, Inc., CA, USA) with a cartridge (Model No. DMC-11610) was used to print the pattern of nanoparticle structure on the substrate. The printer head has 16 nozzles in a row with a spacing of 254 μm. Each nozzle is approximately 21.5 μm in diameter and supports 10 pL droplets. The operation of each nozzle is independent of each other. The center-to-center droplet spacing ranged from 5 to 254 μm and was adjusted by changing the angle of the printer head in one-micron increments and was dependent upon the dpi settings. A droplet spacing of 200 µm was primarily used in all experiments to maintain an appropriate distance, and thus, prevented the overlapping of injected droplets^30,38^.

The wafer (LG Siltron Inc., South Korea) was used as a substrate. The full wafer was cut to the required dimension of 7 cm × 3 cm for printing. The substrate was treated by chlorotrimethylsilane in vacuum for 45 min prior to inkjet printing. In order to prepare the ink, a 1000 µL solution of mono-disperse silica nanoparticles (20% w/v) with diameters of 500 nm (Polysciences, Warrington, USA) was centrifuged at 4000 rpm for 12 min, and the solution was then removed to obtain a highly concentrated nanoparticle suspension (>20% w/v). The nanoparticle suspension was then mixed with formamide (FA) (Sigma Aldrich, Korea) to result in a final concentration of 20% (v/v) to control the evaporation time for better self-assembly and deposition on a substrate. The as-prepared mixed nanoparticle suspension was ultra-sonicated for 10 min to ensure complete dispersion (5510E-DTH, Bransonic, USA), and then injected at a room temperature of 24 °C unless otherwise noted.

### Hydrogel for humidity sensor

The humidity sensor was fabricated using acrylamide. In order to prepare the hydrogel solution, 10 ml solution of acrylamide/Bis-acrylamide(1610114, Bio-Rad, California, USA) was blended with 20 µl solution of 2-hydroxy-2-methylpropiophenone (405655, Sigma Aldrich, Korea), which corresponds to a photoinitiator. The hydrogel solution was poured on the PC and cured using UV. The sample was exposed to ultraviolet light of 250–400 nm at 90 mW/cm^2^ for 15 s. After photopolymerization, the upper hydrogel layer was carefully peeled off using forceps similar to that in PDMS. With respect to the control groups, various upper hydrogel layers were realized using a polyimide spacer (44 μm thickness).

### Characterization of the colloidal crystal–PDMS composite sensor

The periodicity and coverage of colloidal crystal were evaluated by surface imaging by using a scanning electron microscope (SEM) to observe colloidal crystals. We obtained images of the (1) colloidal crystals after crystallization, (2) colloidal crystal–PDMS composite (after NET), and (3) residual PDMS layer. In order to evaluate the sensing ability of the chemical sensor, various organic solvents (ethanol, methanol, 2-propanol, acetone, benzene, toluene, and xylene) were introduced on the surface and optical properties were measured. The swelling ratio of PDMS depends on the polarity of the solvent, and thus, experiments were performed by dividing the same into two groups. Based on the results of a previous study^9^, the expansion capacity of the solvent increased in the following order: methanol, ethanol, acetone, benzene, toluene, and xylene. A solvent with a relatively low PDMS expansion ratio (ethanol, methanol, and acetone) was directly introduced on the apparatus surface. We measured the optical properties of each device with different thicknesses by using a spectrometer (USB 2000, Ocean Optics). With respect to the BTX (benzene, toluene, and xylene) solvents with high expansion ratios, it was difficult to measure the color change when the NET-processed sensor was directly exposed. Therefore, the devices were tested to detect different vapor phase analytes at a room temperature of 24 °C and atmospheric pressure. In order to produce different analyte concentrations, saturated vapor (124,720 ppm for benzene, 38,130 ppm for toluene, and 11,220 ppm for xylene) was first generated by bubbling nitrogen through a wash bottle that contained the analyte in a liquid phase^39^. A two-channel mixing flowmeter (RK1202M, Kofloc, Japan) was then used to dilute back the saturated vapor with pure nitrogen to lower the analyte concentration.

## Electronic supplementary material

Supplementary information

supplementary video1

supplementary video2

supplementary video3
