# Supplementary material for: Ultra-fast responsive colloidal–polymer composite-based volatile organic compounds (VOC) sensor using nanoscale easy tear process
Source: Sci Rep. 2018 Mar 28;8:5291. doi: 10.1038/s41598-018-23616-8 (PMC5871848; doi:10.1038/s41598-018-23616-8)
Supplement: Supplementary file 1 — Supplementary information [file 41598_2018_23616_MOESM1_ESM.doc]

Supplementary Information

Ultra-fast responsive colloidal–polymer composite-based volatile organic compounds (VOC) sensor using nanoscale easy tear process

Hyung-Kwan Chang^a^, Kyutae Jang^a^, Ashish K. Thokchom^b^, Taesung Kim^b^, and Jungyul Park^†a,c^

^a^Department of Mechanical Engineering, Sogang University, 35 Baekbeom-ro (Sinsu-dong), Mapo-gu, Seoul 04107, Korea

^b^ Department of Mechanical Engineering, Ulsan National Institute of Science and Technology (UNIST), 50 UNIST-gil, Ulsan 44919, Republic of Korea

^c^Interdisciplinary Program of Integrated Biotechnology, Sogang University, 35 Baekbeom-ro (Sinsu-dong), Mapo-gu, Seoul 04107, Korea

^†^Corresponding author. Tel.: +82 2 705 8642; fax: +82 2 701 7075.

E-mail address: sortpark@sogang.ac.kr (J. Park)

† Electronic supplementary information (ESI) available:

**Simulation model**

Structural dynamics simulations were performed by COMSOL to predict the initial cracks in the colloidal crystal–PDMS composite. The simulation model consists of three layers of nanoparticles and the surrounded PDMS. The Young’s modulus and Poisson’s ratio of PMDS used in the simulation were 2.05 MPa and 0.49, respectively, and those of polystyrene 3200 MPa and 0.42, respectively. The fixed constraint was applied at the bottom boundary and the parametric sweep was carried out to invoke the prescribed displacement (normal and shear direction) of the upper boundary until the ultimate tensile strength of the PDMS occurred. According to the interaction pair potential proposed by vein Mie, the van der Waals force (attractive adhesion) was maximized at a distance of 0.4 nm[^1^](#_ENREF_1). Therefore, the initial inter-particle distance was defined as 0.4 nm. The van der Waals force is expressed as a function of the inter-particle distance and is defined using the Hamaker constant between the two particles and between the particle and the flat surface, as shown in Fig. S1(a). Where *A* denotes the Hamaker constant (10^-19^ J), *R* denotes the radius of the particle (110 nm), *D* denotes the inter-particle distance, and u_i_ denotes the displacement of the particle. After the simulation, post-processing was performed and the von Mises stress plot was obtained.

In the real situation of peeling-off, the bulk PDMS was pulled by forceps and it induces shear stress at the boundary naturally. To mimic this situation, another simulation model for the boundary position was developed, which was similar to the previous one. The different point is that the symmetric boundary condition was imposed at one side, and the free boundary at the other side. Then, the normal displacement was applied to bulk PDMS. Of course, the middle position of colloidal crystal-PDMS composite corresponds to the previous simulation model under the normal displacement (Fig. 2(b)). As shown in Fig. S1(b), the concentrated stress was found at the first layer of colloidal crystal-PDMS, which is similar to the simulation results under shear deformation.

**References**

1. J. N. Israelachvili, *Intermolecular and surface forces*, Academic Press, Burlington, MA, 3rd edn., 2011.


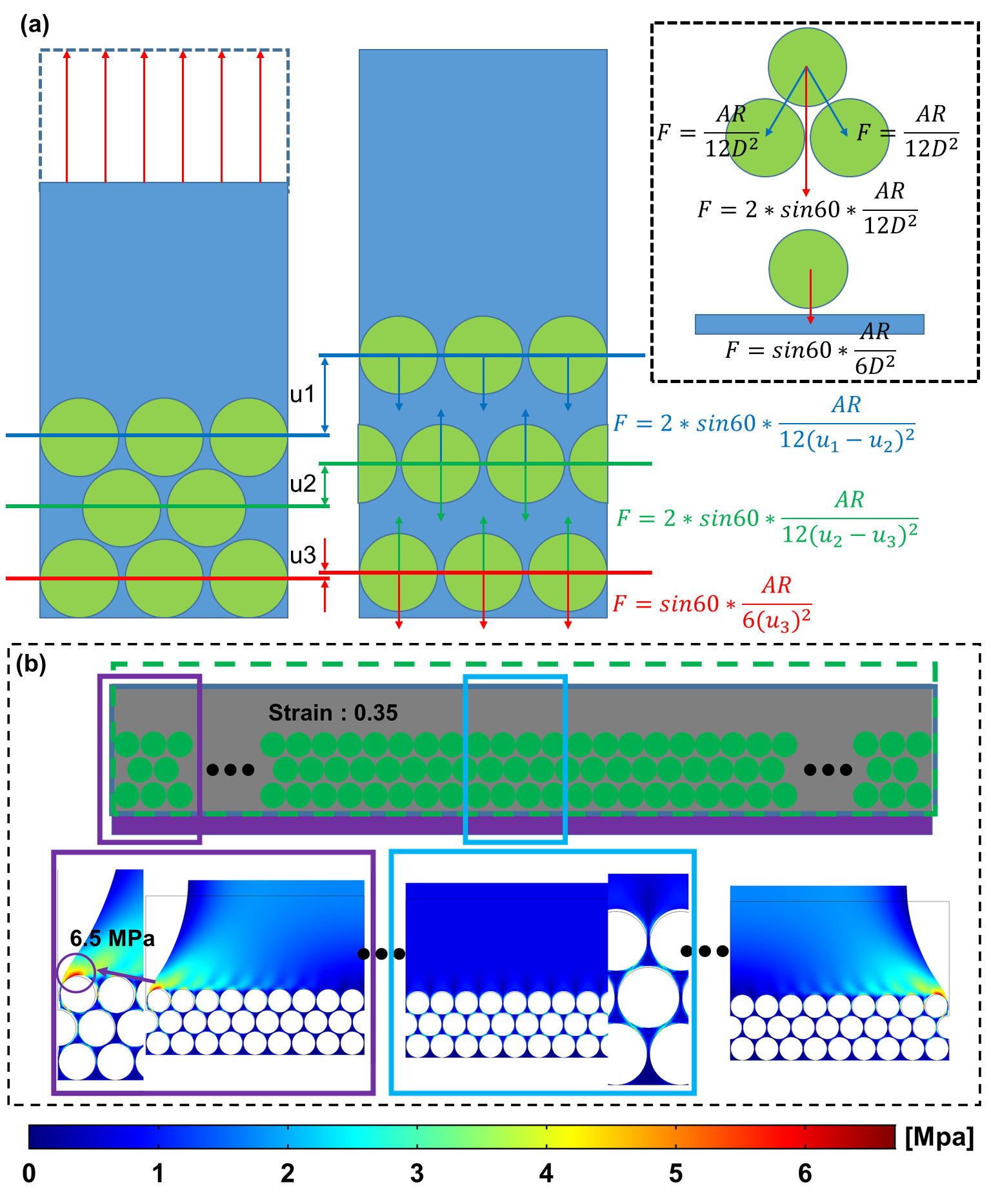


**Figure S1**. (a) Schematic of the simulation model and boundary conditions including the van der Waals force. (b) Simulation models for mimic the real peel-off by pulling the bulk PDMS with a forceps.


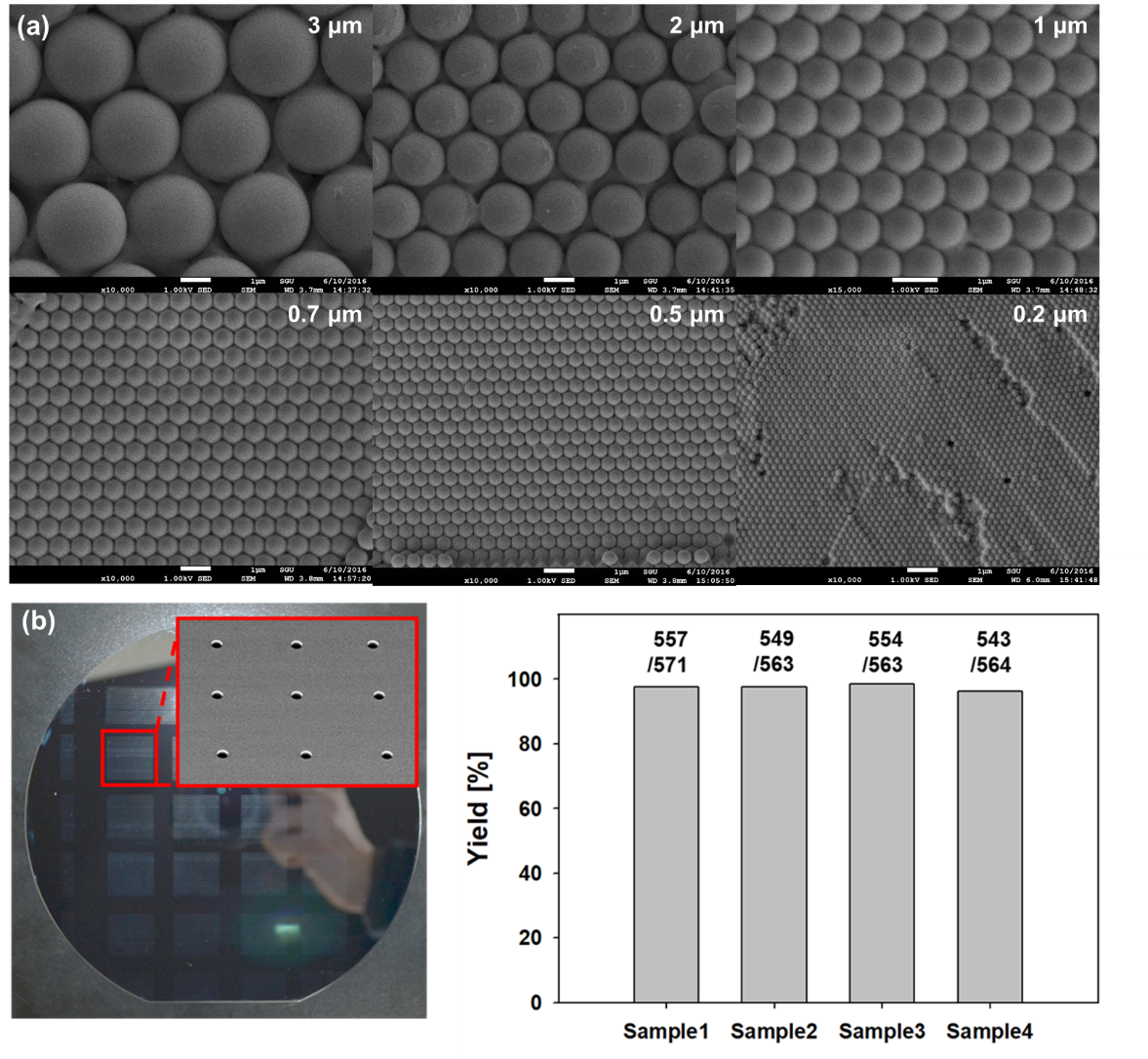


**Figure S2**. (a) SEM image of NET-processed colloidal crystal–PDMS composite relative to various particle sizes. (b) Dome-shaped colloidal crystal arrays constructed on wafers and successful yield of NET process.


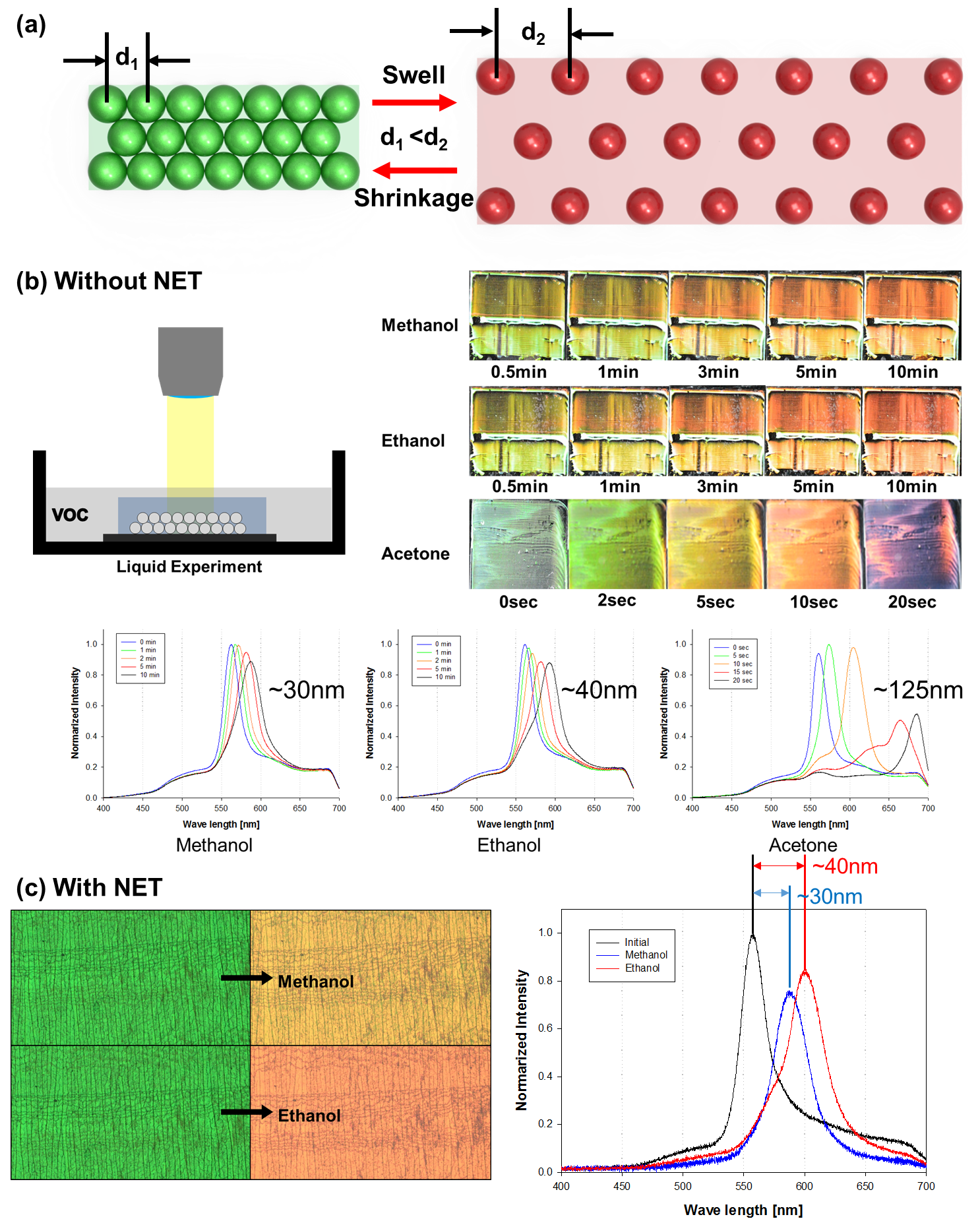


**Figure S3**. (a) Detection principle of the colloidal crystal-PDMS composite chemical sensor. Color change and bandgap shift with respect to the colloidal crystal–PDMS composites (b) without NET process and (c) with NET process when exposed to different types of organic solvents.

**
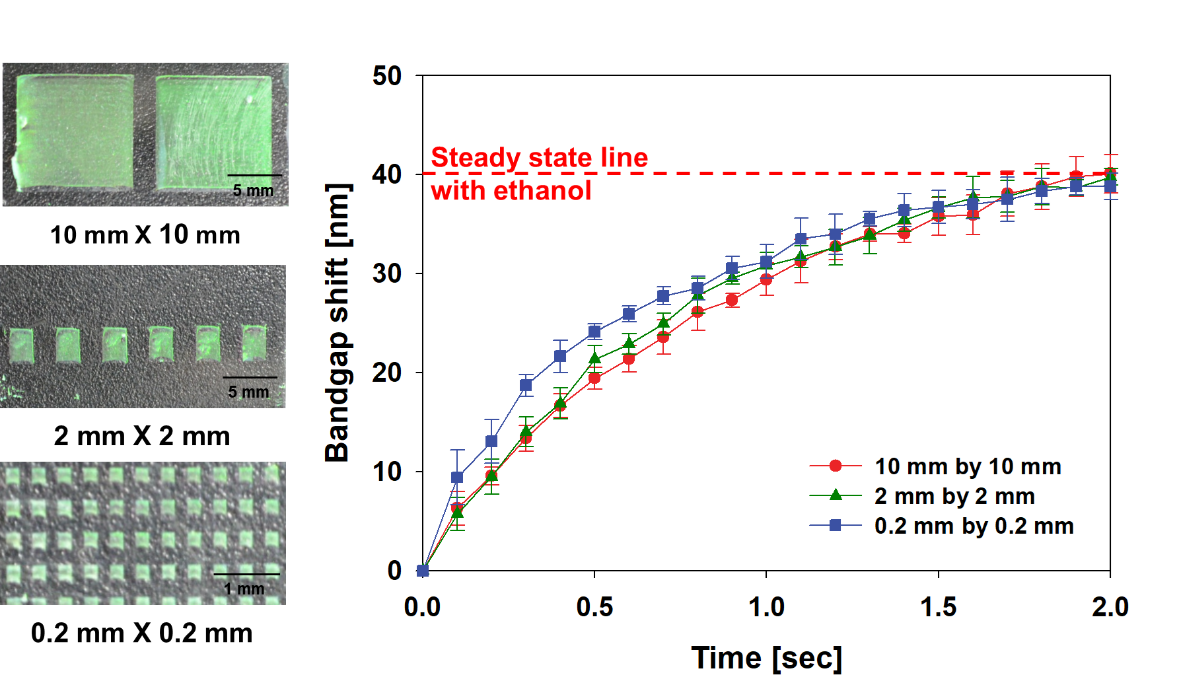
**

**Figure S4**. Response time results depending on pattern size


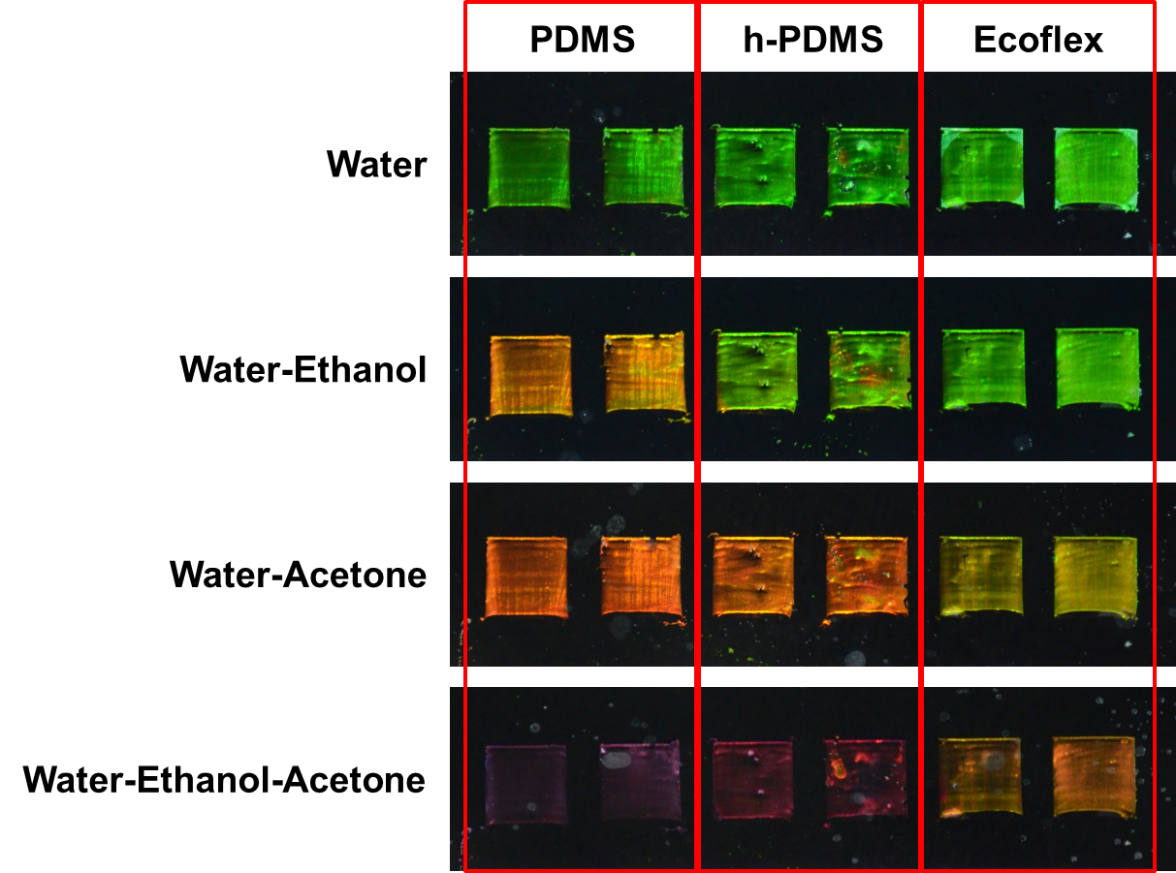


**Figure S5**. Color change results of a 6 × 1 array pattern made of different polymers
